# Spatial transcriptomics reveals gene interactions and signaling pathway dynamics in rat embryos with anorectal malformation

**DOI:** 10.1007/s10565-024-09878-1

**Published:** 2024-05-21

**Authors:** Chen-Yi Wang, Mu-Yu Li, Si-Ying Li, Xiao-Gao Wei, Zheng-Wei Yuan, Xiao-Bing Tang, Yu-Zuo Bai

**Affiliations:** 1 Department of Pediatric Surgery, Shengjing Hospital of China Medical University, Sanhao Street No. 36, Shenyang, 110004 Liaoning China; 2 Key Laboratory of Health Ministry for Congenital Malformation, Shengjing Hospital of China Medical University, Shenyang, Liaoning China

**Keywords:** Anorectal malformation, Spatial transcriptome, Cloacal development, Hindgut, PROGENy

## Abstract

Anorectal malformation (ARM) is a prevalent early pregnancy digestive tract anomaly. The intricate anatomy of the embryonic cloaca region makes it challenging for traditional high-throughput sequencing methods to capture location-specific information. Spatial transcriptomics was used to sequence libraries of frozen sections from embryonic rats at gestational days (GD) 14 to 16, covering both normal and ARM cases. Bioinformatics analyses and predictions were performed using methods such as WGCNA, GSEA, and PROGENy. Immunofluorescence staining was used to verify gene expression levels. Gene expression data was obtained with anatomical annotations of clusters, focusing on the cloaca region's location-specific traits. WGCNA revealed gene modules linked to normal and ARM cloacal anatomy development, with cooperation between modules on GD14 and GD15. Differential gene expression profiles and functional enrichment were presented. Notably, protein levels of Pcsk9, Hmgb2, and Sod1 were found to be downregulated in the GD15 ARM hindgut. The PROGENy algorithm predicted the activity and interplay of common signaling pathways in embryonic sections, highlighting their synergistic and complementary effects. A competing endogenous RNA (ceRNA) regulatory network was constructed from whole transcriptome data. Spatial transcriptomics provided location-specific cloaca region gene expression. Diverse bioinformatics analyses deepened our understanding of ARM's molecular interactions, guiding future research and providing insights into gene regulation in ARM development.

## Introduction

Congenital anorectal malformation (ARM) represents one of the most common congenital gastrointestinal anomalies in pediatric surgery and is a globally monitored congenital disorder by the World Health Organization. Its incidence in neonates ranges from approximately 1 in 2000 to 1 in 5000, with a slightly lower prevalence among females than males (Kapapa et al. 2021; Theron et al. 2015). ARM arises from developmental disturbances in the embryonic hindgut and is often inherited through various modes, including autosomal dominant or recessive patterns and multifactorial inheritance (Falcone Jr et al. 2007; Kause et al. 2019). Human ARM manifests as a spectrum of structural abnormalities, such as anal atresia and rectourethral fistulas, resulting from defects in the development of the cloaca during the 4–8th weeks of gestation (Moore 2013; Zhang et al. 2011). Clinical sampling during this critical period is exceptionally challenging. Previous research has demonstrated that ethylenethiourea (ETU) induction in rat embryos at gestational days (GD) 14–16 leads to anomalies in cloacal development, closely resembling the human rectourethral fistula. Consequently, the ETU-induced rat ARM model has become a widely used approach for ARM research (Martins et al. 2019; Macedo et al. 2007). Given the intricate pathological alterations in ARM, the precise molecular mechanisms underlying its pathogenesis remain elusive (Wang et al. 2015). Surgical intervention, although essential for anatomical reconstruction, cannot reverse the molecular alterations that trigger these malformations (Iwai and Fumino 2013). Therefore, a comprehensive investigation into the molecular mechanisms of ARM holds significant promise for prenatal screening and exploring novel early diagnostic and therapeutic strategies.

Previous studies have conducted whole transcriptome sequencing of cloacal tissues from normal and ARM rat embryos to identify key genes (Li et al. 2021; Xiao et al. 2018). However, the cloacal tissue comprises numerous intricate anatomical structures, making it challenging to associate gene expression data with specific anatomical locations using a whole-tissue sequencing approach. The 10×Genomics Visium spatial transcriptomics technology, which combines microscopy and RNA sequencing, allows the integration of spatially resolved mRNA expression data with stained tissue sections (Shen et al. 2022). This technology enables the generation of high-throughput transcriptomic data from entire tissue sections. In this study, we applied spatial transcriptomic analysis to sequence tissue sections from GDs 14–16 normal (referred to as N14–16) and ARM (referred to as A14–16) rat embryos. These sections were annotated based on their anatomical structures, resulting in the identification of eight clusters (clusters 0–7) across six tissue sections. These clusters corresponded to specific anatomical regions, including the urethra, hindgut, bladder, urorectal septum (URS), genital tubercle, vertebral bodies, neural tube, and abdominal small intestine.

This study aimed to analyze the relevant gene data mainly from the cloacal region of normal and ARM rat embryos at GDs 14–16 obtained using spatial transcriptomics technology. We present gene modules associated with different GDs and anatomical structures, illustrate expression patterns of select differentially expressed genes, conduct enrichment analyses, explore the relevance and activities of common signaling pathways in the cloacal region, and construct a competing endogenous RNA (ceRNA) regulatory network through integrated whole transcriptome sequencing. Investigating and analyzing the gene expression data obtained from spatial transcriptomics will deepen our understanding of the gene regulatory interactions during the process of ARM development.

## Materials and Methods

### Preparation of ARM animal models and embryo embedding

Wistar rats, aged 10–12 weeks and weighing 200–230 g (provided by Beijing HFK Bioscience Co., Ltd.), were raised in a specific pathogen-free (SPF) facility. Sexually mature and healthy female Wistar rats were selected, and they were co-housed with the male rats at a 3:1 ratio overnight. The presence of sperm in the vaginal smears of female rats on the following morning was recorded as GD0. Pregnant female rats were housed separately and randomly assigned to either the ARM group or the normal group. On GD10, pregnant rats in the ARM group received an oral gavage of 1% ETU (Sigma-Aldrich; Merck Millipore, Darmstadt, Germany) at a dose of 125 mg/kg, while pregnant rats in the normal group received an equivalent volume of normal saline. Anesthesia was induced using isoflurane inhalation followed by an intraperitoneal injection of lidocaine on GDs 14–16, and cesarean sections were performed to collect the embryos. A total of 10 pregnant rats were collected for the normal group, comprising 4 pregnant rats at N14 (45 embryos), 3 at N15 (39 embryos), and 3 at N16 (32 embryos). For the ARM group, a total of 13 pregnant rats were collected, with 6 pregnant rats at A14 (62 embryos), 4 at A15 (40 embryos), and 3 at A16 (29 embryos). The embryos were washed with pre-cooled sterile normal saline to eliminate surface blood. Sterile gauze was used to dry any remaining liquid. Subsequently, the embryos were submerged in an embedding box filled with pre-chilled optimal cutting temperature compound (OCT; SAKURA, Japan). They were oriented horizontally in a sagittal position and rapidly immersed in pre-chilled isopentane (Sigma-Aldrich) for 1 min. Once the OCT was fully solidified, the samples were stored in a -80 °C freezer.

### Slide preparation

Spatial transcriptomics slides were designed with four identical capture areas, each measuring 6.5×6.5 mm, and housing 5,000 spots containing barcoded primers supplied by 10×Genomics. These primers were attached to the slide at their 5′ end and included a cleavage site, a T7 promoter region, a partial read1 Illumina handle, a unique spatial barcode specific to each spot, a unique molecular identifier (UMI), and a 20(T) VN sequence. The spots, each with a diameter of 55 μm, were organized in a regularly spaced hexagonal grid, ensuring that each spot was surrounded by six other spots, with a center-to-center distance of 110 μm. Frozen tissue sections, cut to a thickness of 10 μm using a pre-cooled cryostat, were precisely positioned onto pre-chilled Visium Tissue Optimization Slides (10×Genomics, Cat#3000394) and Visium Spatial Gene Expression Slides (10×Genomics, Cat#2000233). These prepared slides were subsequently stored at –80 °C until further use.

### Tissue optimization

The spatial transcriptomics protocol was meticulously adapted to suit the tissue under investigation, following established guidelines (Berglund et al. 2018). In summary, several adjustments were made to the staining procedure. This involved the exclusion of isopropanol, reducing the incubation time for hematoxylin and bluing buffer, and augmenting the eosin concentration. Furthermore, we determined the ideal permeabilization incubation time through experimentation with the Visium Tissue Optimization Slides. To enhance tissue removal in the one-step protocol, a higher ratio of proteinase K to PKD buffer was implemented. Once the optimal conditions were ascertained, we proceeded by cutting three cryosections per sample at a thickness of 10 μm onto spatial slides and conducting immediate processing.

### Fixation, staining, and imaging

The slides underwent a series of procedures: incubation at 37 °C for 1 min, fixation in paraformaldehyde for 30 min, and subsequent washing in 1×PBS. Staining involved incubation with Mayer’s hematoxylin (Dako, Agilent, Santa Clara, CA) for 4 min, bluing buffer for 30 s, and eosin (Sigma–Aldrich) diluted 1:5 in Tris-base for 30 s. RNase-free water washed the slides after each staining step. Following air-drying, mounting occurred with 85% glycerol (Merck Millipore, Burlington, MA) and coverslips (Menzel-Glaser). Bright-field images at 20× magnification were captured using the Metafer Slide Scanning platform. For pre-permeabilization, sections were incubated with collagenase and bovine serum protein at 37 ° C for 20 min and finally infiltrated with 0.1% pepsin for 7 min.

### Reverse transcription (RT), spatial library preparation, and sequencing

RT was conducted as previously described (Berglund et al. 2018). Following RT, the wells were rinsed with 0.1× SSC and subjected to incubation at 56 °C with periodic shaking for 1.5 hours using a mixture of Proteinase K (QIAGEN) and PKD buffer (QIAGEN, pH 7.5) at a 1:1 ratio for tissue removal. The spatially barcoded cDNA was enzymatically released as outlined in previous descriptions. The resulting supernatants containing released cDNA were gathered and transferred to 96-well plates for the preparation of spatial transcriptomics libraries using an automated MBS 8000 system (Jemt et al. 2016). In short, the process involved the synthesis of the second-strand cDNA, followed by *in vitro* transcription, adaptor ligation, and a subsequent round of RT. Sequencing handles and indices were incorporated during an indexing PCR, and the finalized libraries were purified and quantified using the methods outlined in a prior study (Ståhl et al. 2016). Sequencing was performed on the Illumina NovaSeq 6000 with a sequencing depth of at least 50,000 reads per spot and 150 bp (PE150) paired-end reads (performed by Biomarker Technologies Corporation, Beijing, China).

### Spot visualization and image alignment

The process of spot staining and imaging was previously detailed. In summary, fluorescently labeled probes were employed to stain primer spots through hybridization, and these spots were subsequently captured using the Metafer Slide Scanning platform. The resulting spot image, along with the earlier acquired bright-field tissue image of the corresponding region, was loaded into the web-based Spot Detector tool for spatial transcriptomics. (Wong et al. 2018). The alignment of the two images took place, and the integrated tissue recognition tool was utilized to isolate spots covered by tissue.

### Weighted Gene Co-expression Network Analysis (WGCNA)

WGCNA analysis was conducted using the R packages, hdWGCNA (version 0.1.2.1; https://smorabit.github.io/hdWGCNA/index.html) and WGCNA (version 1.71; https://bmcbioinformatics.biomedcentral.com/articles/10.1186/1471-2105-9-559).

The *SetupForWGCNA* function was used to set the name of the hdWGCNA experiment, the *MetacellsByGroups* function was used to construct the metacell expression matrix of the single-cell dataset, and the *NormalizeMetacells* function was used to normalize the cell expression matrix. The *SetDatExpr* function was used to specify the expression matrix to be used for network analysis, with the default being the expression matrix of metacells. The *TestSoftPowers* function was used to guide the construction of the soft threshold for the co-expression network, simulating the similarity between the co-expression network and the scale-free graph under different soft power thresholds. The *PlotSoftPowers* function was used to visualize the result of the parameter sweep. A threshold of ≥ 0.8 was generally selected for fitting the scale-free topology model. The *PlotDendrogram* function was used for the visualization of the co-expression network, and the *ModuleEigengenes* function was used to calculate the module eigengenes in individual cells. The ME matrix had one row for each cell and one column for each module. The *GetMEs* function was used to extract this matrix from the Seurat object. The *ModuleConnectivity* function was used to calculate the kME value, and the *PlotKMEs* function was used for data visualization. The analysis of key module genes was based on the fact that genes in the same module usually have similar functions. The feature genes of the module were analyzed using dimensionality reduction, and the connectivity of each gene was calculated based on the feature genes to identify the highly connected hub genes in the module and to visualize them for display.

### Immunofluorescence staining

Paraffin sections, providing a clear and comprehensive view of the urethra, hindgut, URS, and rectourethral fistulas, were chosen for staining. Subsequently, the sections underwent deparaffinization and antigen retrieval. Blocking was carried out with serum (ZSGB-BIO, Beijing, China) at 25 °C for 1 hour, followed by overnight incubation at 4 °C with primary antibodies. The primary antibodies used were as follows: Anti-Pcsk9 (1:100), anti-Hmgb2 (1:100), and anti-Sod1 (1:100), all sourced from Proteintech (Wuhan, China). After PBS washes, the sections were incubated at 25 °C in the dark for 1 hour with fluorescent secondary antibodies: goat anti-rabbit 488 (1:100; Proteintech). Following PBS washes, the sections were stained with DAPI solution (Servicebio Technology, Wuhan, China) for 10 minutes. Finally, the sections were mounted using an anti-fluorescence fading medium (Solarbio, Beijing, China), and images were captured using a laser-scanning confocal microscope (LSM 880, Zeiss, Oberkochen, Germany).

### Pathway RespOnsive GENes (PROGENy) and non-negative matrix factorization (NNMF)

We conducted pathway activity analysis using the PROGENy package (version 1.12.0) in R software (version 4.0.3). To standardize spatial transcriptomic data, we set the PROGENy standardization parameter to FALSE and used the ScaleData function from the Seurat package for normalization. After enriching the spatial transcriptome data with PROGENy activity scores, we scaled the data to obtain spot-specific scores. These activity scores were then integrated with the clustering results, and we computed pathway-specific mean activity scores for each cluster. This dataset was used to create a heatmap with the R package heatmap. To reduce dimensionality, we employed the NNMF approach with assistance from the STutility R package. We assessed the correlation between PROGENy activity scores and dimensionality reduction outcomes using Pearson's method and visualized it with a correlation heatmap using the R package heatmap.

### Whole transcriptome joint analysis

Using the R function, *phyper*, the P-value for ceRNA pairs was calculated using the hypergeometric distribution test. The *p.adjust* function (selecting the FDR method) was the applied to correct the P-values, with a filter set to remove some ceRNA pairs that did not meet the standard with hypergeometric test P-values < 0.01 and FDR values < 0.01. Co-expressed relationships were identified by calculating the correlation between two types of RNA, and positively correlated ceRNA pairs with a correlation coefficient greater than 0 were selected based on co-expression relationships. Seurat (version 4.1.0) was used to screen for differentially expressed genes in the spatial transcriptome, with the most relaxed parameter as P-value < 0.05 and FC > 1.5. The edgeR package (version 3.32.1) for the whole transcriptome was used for differential parameter modulation, with the most relaxed parameter as P-value < 0.05 and FC > 1.5. Finally, Cytoscape software was used to visualize and sort the connectivity of the ceRNA network. The data produced for the ceRNA network are available on NCBI GEO datasets (Track No. GSE159306). To access the data, please visit https://www.ncbi.nlm.nih.gov/geo/query/acc.cgi?acc=GSE159306.

## Results

### WGCNA reveals gene modules associated with the cloacal region

#### Construction of adjacency matrix and module division

**Fig. 1:**
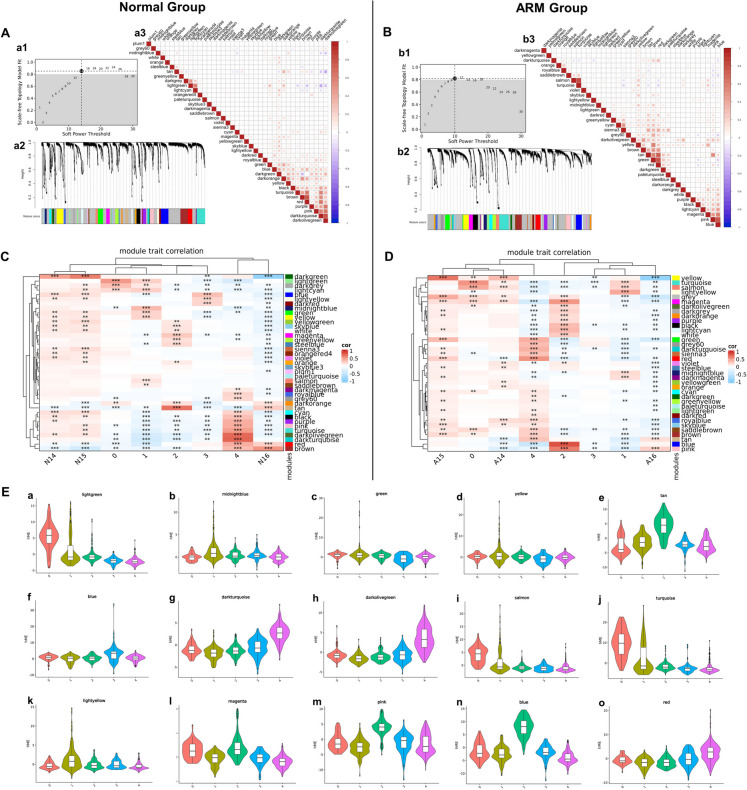
WGCNA shows the gene modules related to the anatomic structure of normal and ARM cloacal regions. **A.** a1, the optimal soft power threshold for N14–16 samples; a2, gene co-expression network diagram of N14–16 samples, including gene hierarchical clustering tree and gene module color division; a3, module intercorrelation heat map based on MEs matrix drawn for N14–16 samples. **B.** b1, the optimal soft power threshold values for A14–16 samples; b2, gene co-expression network diagram of A14–16 samples, including gene hierarchical clustering tree and gene module color division; b3, module intercorrelation heat map based on MEs matrix drawn for A14–16 samples. **C.** Module-trait correlation heat map based on MEs matrix drawn for N14–16 samples. **D.** Module-trait correlation heat map based on MEs matrix drawn for A14–16 samples. **E.** Violin plots showing the correlation between gene color modules and clusters 0–4. Abbreviations: ARM, anorectal malformations; A, anorectal malformations group; MEs, module eigengenes; N, normal group; WGCNA, Weighted Gene Co-expression Network Analysis

WGCNA is a common method used to construct gene co-expression networks (Langfelder and Horvath 2008). Each node represents a gene, and genes with common expression patterns across different samples are grouped into the same gene network. The co-expression relationship between genes is measured by expression correlation coefficients. The ‘TestSoftPowers’ function was used to transform the correlation matrix into an adjacency matrix and iteratively determine the optimal Soft Power threshold so that it was as close to a scale-free network as possible, while preserving connectivity information. The optimal Soft Power threshold for N14–16 samples was 14 (Fig. 1Aa1), and this value for A14–16 samples was 10 (Fig. 1Bb1). Figure 1Aa2 and 1Bb2 are the co-expression network diagrams of genes, where the first part of the diagram depicts the hierarchical clustering tree of the gene based on the topological overlap dissimilarity measure (TOM), with each line representing a gene and similar genes clustered into one branch, while the vertical distance represents the distance between two genes. The second part shows the results of using dynamic programming algorithms to divide the genes in the first part into different modules, with each module represented by a different color, and genes within the same module usually having similar functions. Grey represents the genes that cannot be grouped into any module and are not included in subsequent analyses. In the N14–16 samples, the genes were divided into 39 color modules, whereas in the A14–16 samples, they were divided into 36 color modules.

#### Correlation analysis based on module eigengene matrix

Module eigengenes (MEs) are indicators used to analyze the gene expression profile characteristics in co-expression modules. After conducting principal component analysis (PCA) on the gene expression matrix subset of each module, the first principal component in the PCA matrix of each module was used as the MEs, and MEs were utilized for correlation analysis. Figure 1 Aa3 and 1Bb3 are inter-module correlation heat maps drawn based on the MEs matrix to screen for statistically significantly related and functionally similar modules. Among them, ‘darkolivegreen’ and ‘darkturquoise’; ‘brown’ and ‘red’ modules exhibited a strong positive correlation in the normal group, whereas ‘pink’ and ‘blue’; ‘salmon’ and ‘turquoise’; ‘green’ and ‘red’ modules exhibited a stronger positive correlation in the ARM group.

Figures 1C and 1D are module-trait correlation heat maps drawn based on the MEs matrix to screen for specific modules statistically significantly related to the phenotype. Results in the normal group show that the color modules related to N14 and N15 had a synergistic relationship and were complementary to N16; the correlation of the gene modules with cluster 2 (bladder) and cluster 4 (genital tubercle) also had complementary characteristics. The ‘darkgreen’ module had the strongest positive correlation with N14 and N15 samples, and the strongest negative correlation with N16 samples; the ‘brown’ module had the strongest negative correlation with N14 and N15 samples, and the strongest positive correlation with N16 samples (Fig. 1C). Combined with the violin plot analysis, it was found that the ‘lightgreen’ module had the strongest positive correlation with cluster 0 (urethra); the ‘midnightblue’ module had the strongest positive correlation with cluster 1 (hindgut); the ‘tan’ module had the strongest positive correlation with cluster 2 (bladder); the ‘blue’ module had the strongest positive correlation with cluster 3 (URS); the ‘darkolivegreen’ and ‘darkturquoise’ modules had the strongest positive correlation with cluster 4 (genital tubercle) (Fig. 1Ea–h). Results in the ARM group indicated that the color modules related to A14 and A15 samples were also basically the same and complementary to A16 samples. The ‘yellow’ module had the strongest positive correlation with A14 and A15 samples, and the strongest negative correlation with A16 samples (Fig. 1D); the violin plot suggests that the ‘salmon’ module had the strongest positive correlation with cluster 0 (urethra); the ‘turquoise’ module also had a strong positive correlation with cluster 0 (urethra); the ‘lightyellow’ module had a strongest positive correlation with cluster 1 (hindgut), and the ‘pink’, ‘blue’, and ‘magenta’ modules had a strong positive correlation with cluster 2 (bladder); the ‘red’ module had the strongest positive correlation with cluster 4 (genital tubercle) (Fig. 1Ei–o).

#### Functional enrichment of core module genes

**Fig. 2:**
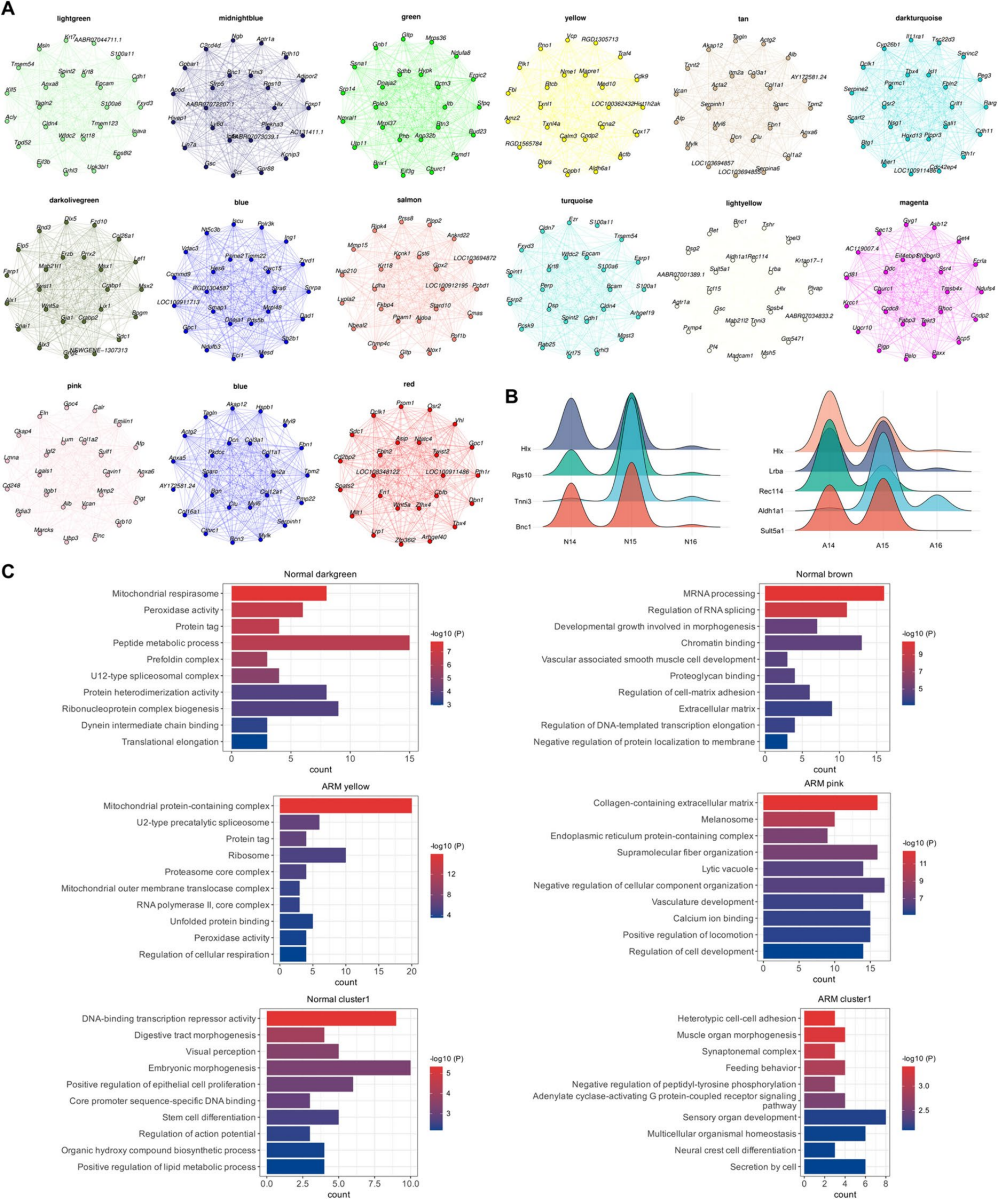
Hub gene network and enrichment analysis. **A.** Network diagram of the top 25 hub genes sorted by kME values. **B.** Ridge map showing the abundance changes of high kME genes in N14–16 and A14–16. **C.** GO enrichment analysis of the gene modules. Abbreviations: A, anorectal malformations group; GO, gene ontology; kME, module eigengene-based connectivity; N, normal group

For each module, hub genes were further explored using the ‘Module Connectivity’ function. The module eigengene-based connectivity (kME) value was calculated to evaluate the effective connectivity between genes. As the default, the top 100 genes with the highest kME values were selected as hub genes, representing the expression trend of the entire module. Additionally, the top 25 hub genes for each module were selected to construct a gene expression network regulatory diagram, showing the mutual regulatory relationships of hub genes in each module (Fig. 2A). The ridge plot indicates the changes in expression abundance of high kME genes in the hindgut region-related genes in the normal and ARM groups. The genes displayed in the N group and A group were only expressed within two GDs, of which, *Hlx* is a gene related to malformations, including congenital diaphragmatic hernia, short bowel, and asplenia (Fig. 2B) (Farrell et al. 2017).

Next, Gene Ontology (GO) enrichment analysis was performed on the top 100 genes with the highest kME values in each module. The ‘darkgreen’ module, which was strongly positively correlated with N14 and N15, was associated with protein metabolism and mitochondrial respiration. The ‘brown’ module, which was strongly correlated with N16, was mainly related to mRNA processing and RNA splicing. The ‘yellow’ module, which was strongly positively correlated with A14 and A15, was associated with mitochondrial protein complexes. The ‘pink’ module, which was strongly correlated with A16, was mainly related to intracellular components. In the gene modules strongly related to the hindgut, those in the normal group were mainly related to digestive tract development and epithelial cell proliferation, whereas those in the ARM group were mainly related to cell secretion and homeostasis (Fig. 2C).

#### Distribution and enrichment analysis of differentially expressed genes

**Fig. 3:**
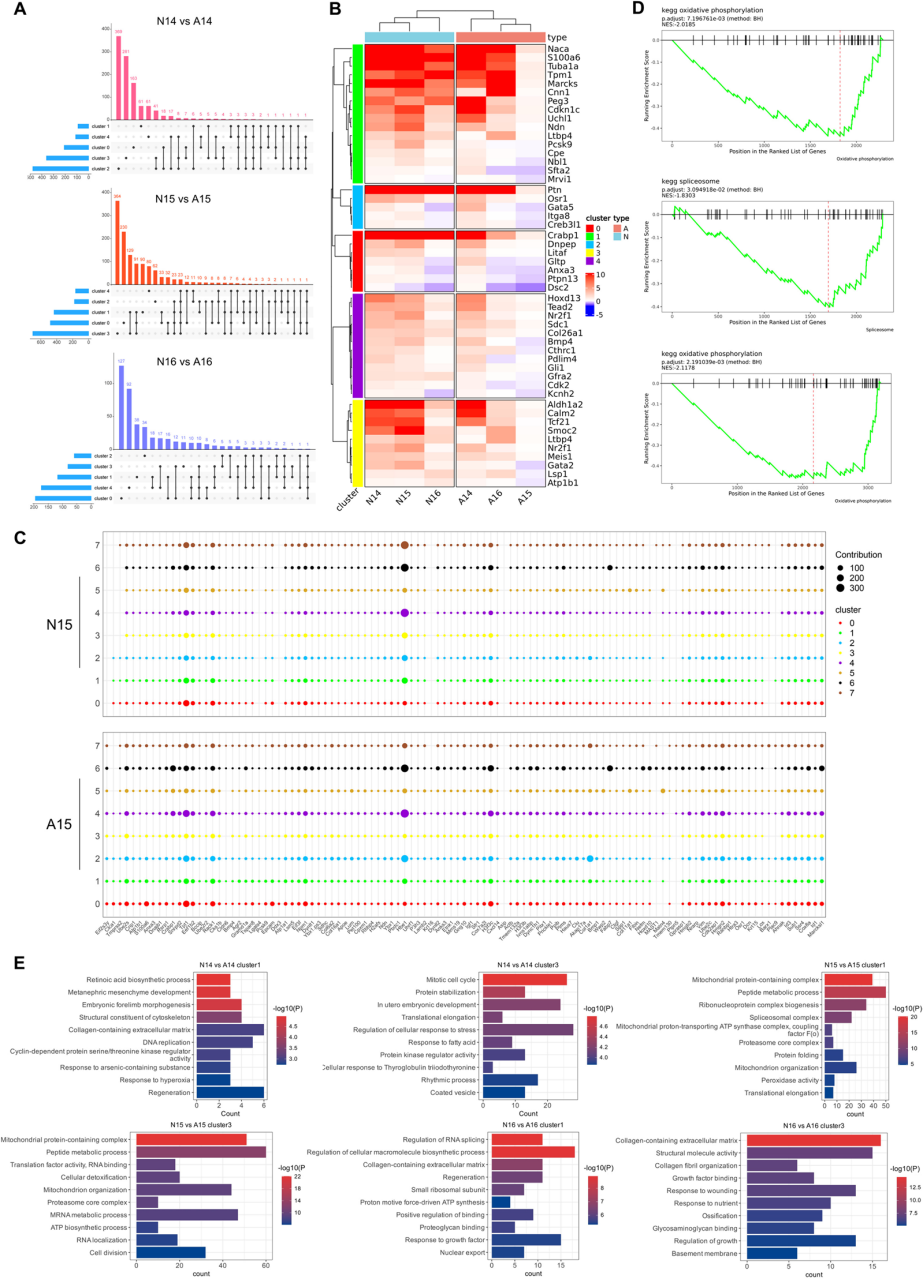
Differentially expressed gene data display and enrichment analysis. **A.** UpSet plot showing the intersection of differentially expressed genes in clusters 0–4. **B.** The heatmap illustrates the expression patterns of selected differentially expressed genes within clusters 0–4 among the six samples. **C.** The dot plot showcases the expression patterns of specific genes within the 8 clusters of GD15. The size of the dots corresponds to the expression abundance. **D.** GSEA analysis of differentially expressed genes in clusters 1 and 3 at GD15. **E.** GO enrichment analysis of differentially expressed genes in clusters 1 and 3. Abbreviations: ARM, anorectal malformations; A, anorectal malformations group; GD, gestational day; GO, gene ontology; GSEA, GeneSet Enrichment Analysis; N, normal group

We set an absolute value of log_2_FC ≥ 0.58 and P < 0.05 as the criteria for differential gene selection. The UpSet plot illustrates the intersections of differentially expressed genes across clusters 0–4 at GDs 14–16. When the ARM group was compared with the normal group, differentially expressed genes appeared in each cluster. At GD14, there were no identical differentially expressed genes between cluster 0 (urethra) and cluster 1 (hindgut), whereas at GD15 and GD16, the urethra and hindgut had 33 and 16 identical differentially expressed genes, respectively (Fig. 3A). The heatmap depicts the expression patterns of differentially expressed genes in clusters 0–4 at GDs 14–16 (Fig. 3B). The dot plot displays gene expression abundances in the 8 clusters at GD15 (Fig. 3C). Gene Set Enrichment Analysis (GSEA) results reveal that differentially expressed genes in cluster 1 at GD15 are associated with oxidative phosphorylation and the spliceosome, while those in cluster 3 are linked to oxidative phosphorylation (Fig. 3D). Figure 3E presents the results of GO enrichment analysis for differentially expressed genes in clusters 1 and 3 at GDs 14–16, primarily involving development-related functions and certain kinase-related processes. We conducted a protein-protein interaction (PPI) analysis of differentially expressed genes in the hindgut during GDs 14–16. Genes were ranked based on their betweenness centrality values, and Table 1 presents the genes with higher connectivity.

**Table 1:** Highly connected genes in the hindgut ranked by betweenness centrality values

GD14		GD15		GD16	
Gene	BC-values	Gene	BC-values	Gene	BC-values
Timp1	330	Uba52	8774	Fn1	1809
Col1a1	324	Cct2	7818	Eef1a1	1165
Sdha	300	Itm2b	7337	Atp5b	1034
Eif2s3x	234	Sod1	5227	Gnb2l1	888
Actg2	198	Acta2	4589	Hsp90ab1	764
Tagln	142	Pfdn5	3848	Calr	743
Cdh3	130	Gnb2l1	3698	Anxa2	5509
Cnn1	96	Polr2g	3580	Apoa1	509
Ddx23	90	Gpc3	3310	Uba52	495
Ctps1	88	Atp5l	3308	Alb	479
Nhp2	46	Cdc42	3061	Srsf1	466
Numbl	46	Snrpd2	3058	Tpt1	410
Hdac2	38	Uqcrh	3035	H3f3c	390
Ccna2	34	Col3a1	2955	Igf2	370
Ppif	26	Ssrp1	2886	Nme2	347
Stmn2	20	Rac1	2808	Npm1	328
Nefl	20	Igf2	2737	Eif4a2	297
Tubb3	12	Snrpf	2710	Hmgb2	282
Sncg	12	Rbbp4	2552	Ubb	274
Ubr4	12	Cdh1	2517	Ddx5	239
Aldh1a2	12	Sec61b	2426	Prdx1	235
Wt1	8	P4hb	2408	Ddx39b	200
Phf10	2	Afp	23808	Mylk	190
		Ppib	2270	Fth1	183
		Mrps33	2133	Shfm1	132
		Tagln	2131	App	122
		Sppl3	2060	Atp5e	115
		Aurkb	2042	Hnrnpa1	113
		Ndn	2036	Hnrnph1	112

Abbreviations: *BC* betweenness centrality; *GD* gestational day

### Validation of partial differential gene expressions in the hindgut

**Fig. 4:**
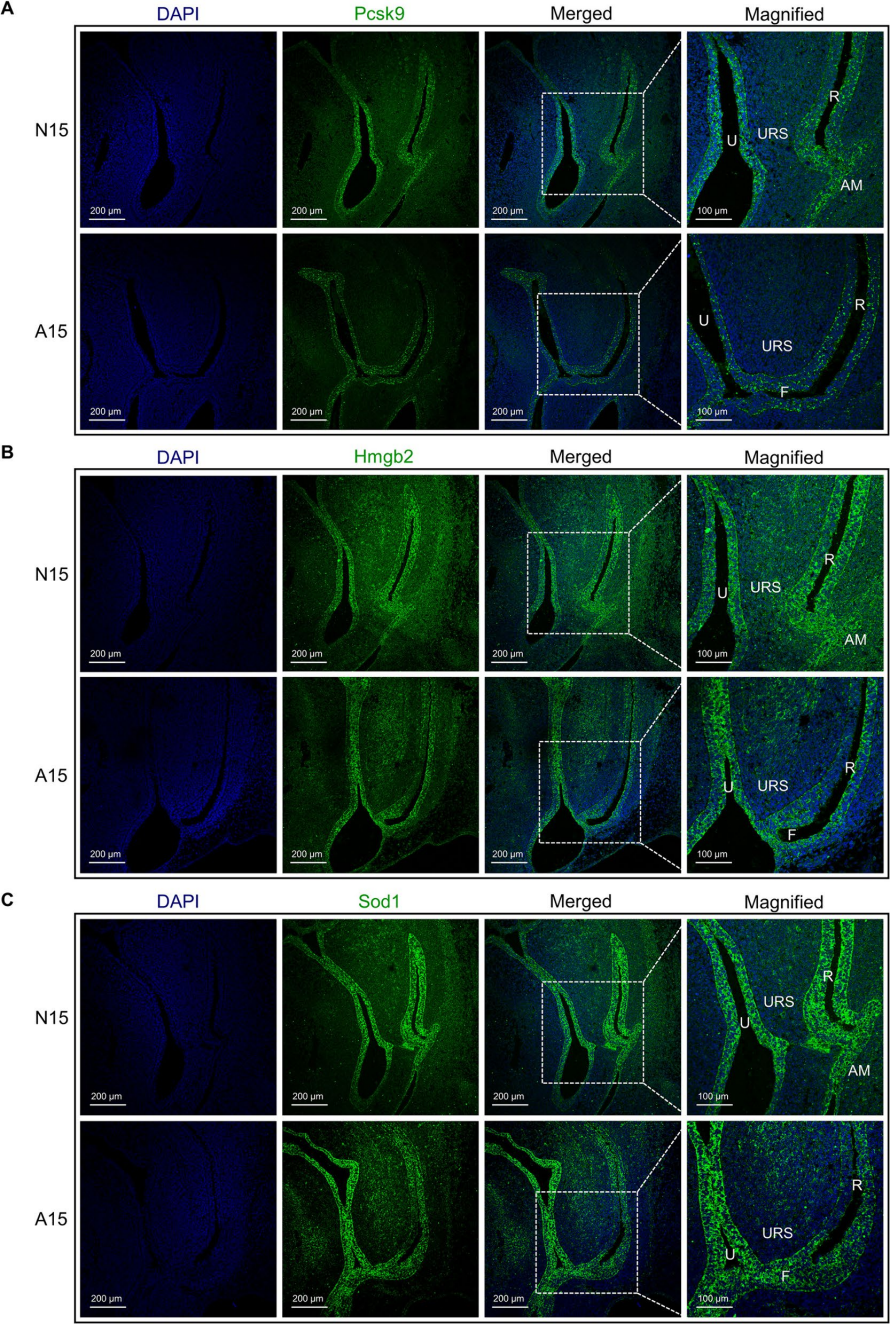
Verification by immunofluorescence staining. **A.** Immunofluorescence staining of Pcsk9 protein in the GD15 hindgut region. **B.** Immunofluorescence staining of Hmgb2 protein in the GD15 hindgut region. **C.** Immunofluorescence staining of Sod1 protein in the GD15 hindgut region. Abbreviations: A, anorectal malformations group; AM, anal membrane; F, rectourethral fistula; N, normal group; GD, gestational day; R, rectum; U, urethra; URS, urorectal septum. Scale bar, 200 μm and 100 μm

We selected three genes, Pcsk9, Hmgb2, and Sod1, which were indicated by spatial transcriptomics to be downregulated in the hindgut of ARM at GD15, for validation of their protein expression using immunofluorescence staining. The results showed that Pcsk9, Hmgb2, and Sod1 were predominantly expressed in the epithelium of the urethra and hindgut in the cloacal region and exhibited significant enrichment in the anal membrane (AM). In A15 slice samples, there was a decrease in fluorescence intensity in the hindgut region, consistent with the sequencing results. Additionally, Pcsk9 showed almost no expression in the URS and surrounding mesenchyme, while Hmgb2 and Sod1 exhibited scattered distribution in the URS and mesenchyme areas (Fig. 4A–C).

### Synergistic and complementary effects among different signaling pathways

**Fig. 5:**
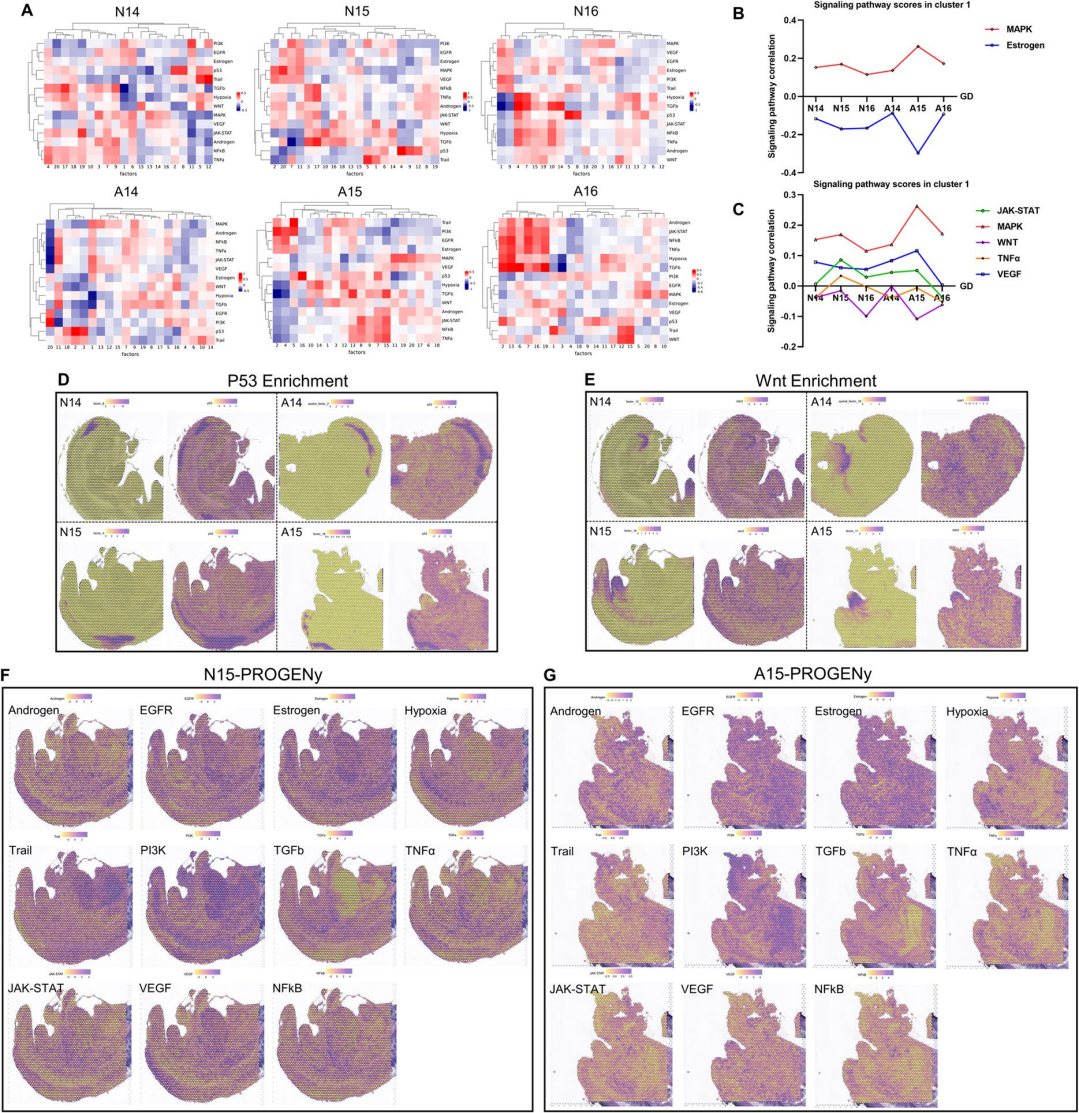
Analysis of signal pathway activity predicted using the PROGENy algorithm. **A.** Heatmap showing the correlation between the activities of 14 signal pathways and NNMF clustering results in six samples. **B.** Trends of correlation changes of MAPK and estrogen signal pathways in cluster 1. **C.** Trends of correlation changes of JAK-STAT, MAPK, Wnt, TNFα, and VEGF signal pathways in cluster 1. **D.** NNMF clustering in the neural tube region and the enrichment localization of the P53 signaling pathway in GD14 and GD15 samples. **E.** Enrichment localization of the Wnt signaling pathway in GD14 and GD15 samples. **F.** Enrichment patterns of common signaling pathways predicted by the PROGENy algorithm in the N15 sample sections. **G.** Enrichment patterns of common signaling pathways predicted by the PROGENy algorithm in the A15 sample sections. Abbreviations: A, anorectal malformations group; GD, gestational day; N, normal group; NNMF, Non-negative Matrix Factorization; PROGENy, Pathway RespOnsive GENes

We used the PROGENy algorithm to predict the activity of signaling pathways through changes in downstream gene expression (Schubert et al. 2018). The PROGENy algorithm covered 14 signaling pathways and was associated with the spots on spatial sequencing slides. We obtained scores for different pathway activities of GDs 14–16 samples in the normal and ARM group. Using NNMF, we obtained 20 unsupervised clustering sets for each sample (Chalise et al. 2020). The heatmap indicates the correlation of 14 signaling pathways in 20 sets, with red indicating a positive correlation and blue indicating a negative correlation. The depth of the color reflects the strength of the correlation (Fig. 5A).

In the urethral and hindgut areas of the six samples, the MAPK signal activity was consistently positively correlated and had the strongest positive correlation with A15. The estrogen signal was consistently negatively correlated with the MAPK signal and had the strongest negative correlation with A15 (Fig. 5B). The negatively correlated Trail signal showed a tendency towards synergistic correlation with the estrogen signal. The correlation between the androgen and estrogen signals showed complementary changes as well as synergistic changes with the MAPK signal in the ARM group. The JAK-STAT, TNFα, VEGF, and MAPK signals showed synergistic correlations; while the Wnt signal showed synergistic correlations with the MAPK signal in the normal group, the opposite was observed in the ARM group, with the correlation difference peaking at GD15 (Fig. 5C).

### Location-specific signaling activity characteristics

The PROGENy algorithm predicted a significant enrichment of the P53 signal in the neural tube region (Fig. 5D). Previous studies have reported increased expression of P53 in neural tube defects (NTDs) and its role in promoting apoptosis (Wu et al. 2013). The Wnt signal was primarily enriched in the tip of the genital tubercle region at GD14 and GD15, with additional enrichment in the hindgut and URS regions (Fig. 5E). Figures F–G illustrate the activity patterns of different signaling pathways in N15 and A15 samples. It is evident that EGFR and PI3K signals show higher enrichment intensity in the urethra and hindgut regions of A15 compared to N15 samples. The VEGF signal exhibits greater enrichment intensity in the genital tubercle region of A15 samples. Furthermore, PI3K and Hypoxia signaling may potentially influence vertebral development.

### Construction of ceRNA regulatory networks using combined whole transcriptome sequencing data

**Fig. 6:**
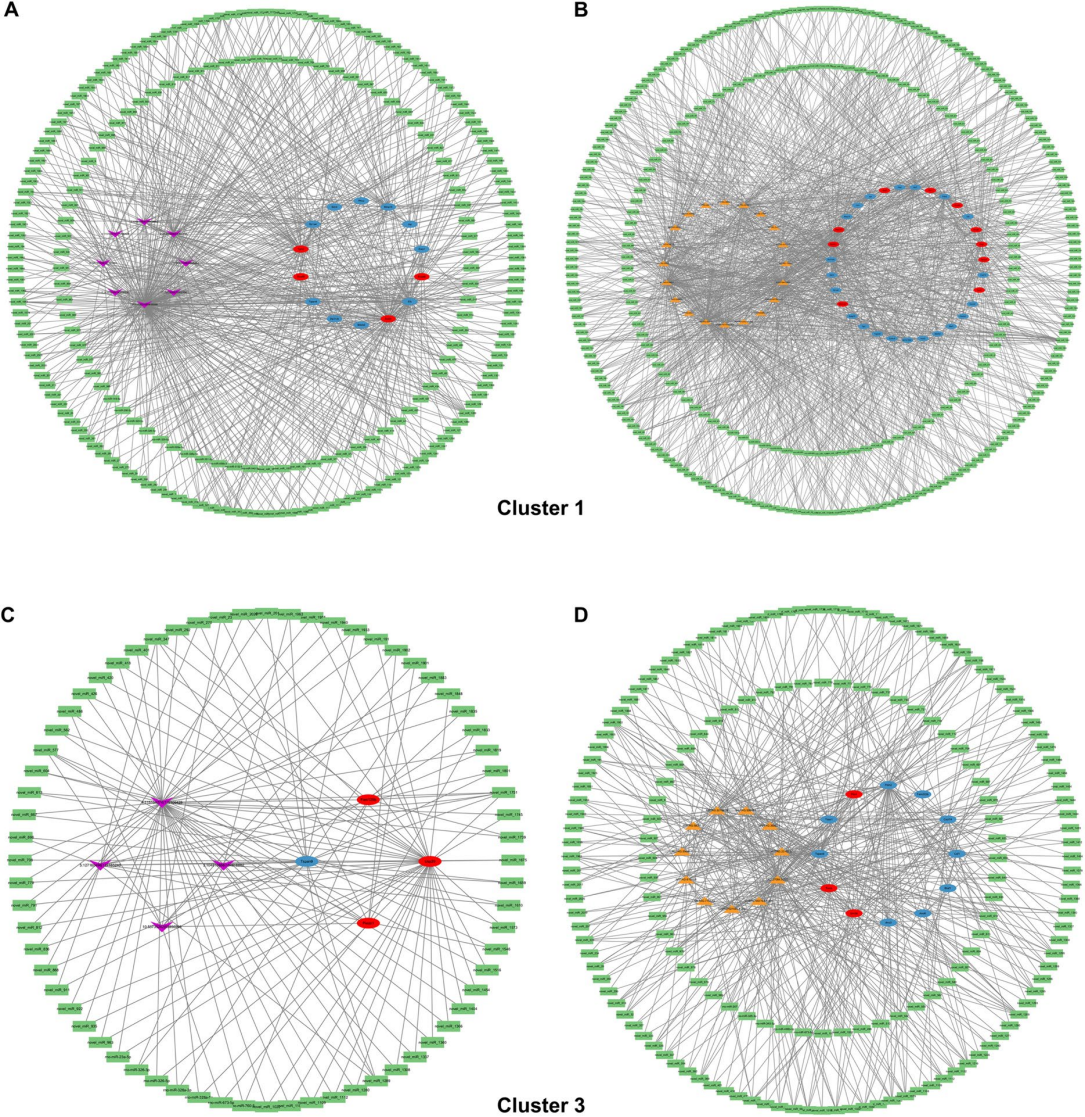
Construction of ceRNA regulatory networks based on joint transcriptome sequencing in the cloacal region. **A.** CircRNA–miRNA–mRNA regulatory network of cluster 1. **B.** LncRNA–miRNA–mRNA regulatory network of cluster 1. **C.** CircRNA–miRNA–mRNA regulatory network of cluster 3. **D.** LncRNA–miRNA–mRNA regulatory network of cluster 3. Purple labels represent circRNA, orange labels represent lncRNA, green labels represent miRNA, red labels represent up-regulated mRNA in the ARM group, and blue labels represent down-regulated mRNA in the ARM group. Whole transcriptome datasets can be queried at https://www.ncbi.nlm.nih.gov/geo/query/acc.cgi?acc=GSE159306 (Track No. GSE159306). Abbreviations: ARM, ARM, anorectal malformations; circRNA, circulating RNA; lncRNA, long non-coding RNA; miRNA, mitochondrial RNA; mRNA, messenger RNA; ceRNA, competing endogenous RNA

The differentially expressed mRNA molecules in clusters 1 and 3 were combined with non-coding RNAs obtained from previous transcriptome sequencing of the cloacal region for prediction, and ceRNA regulatory networks of circular RNA (circRNA) and long non-coding RNA (lncRNA) were constructed (Li et al. 2021). In cluster 1, 8 circRNAs, 223 miRNAs, and 14 mRNAs were constructed into a regulatory network, with the highest connectivity observed for circ53850298, novel-miR-1963, novel-miR-1289, and *Eef2k* (Fig. 6A). Furthermore, 20 lncRNAs, 298 miRNAs, and 31 mRNAs were constructed into a regulatory network, with the highest connectivity observed for lnc127084, novel-miR-1289, and *Rad51c* (Fig. 6B). In cluster 3, 4 circRNAs, 68 miRNAs, and 4 mRNAs were constructed into a regulatory network, with the highest connectivity observed for circ115106428, novel-miR-1751, and *Usp20* (Fig. 6C). Finally, 11 lncRNAs, 167 miRNAs, and 12 mRNAs were constructed into a regulatory network, with the highest connectivity observed for lnc127084, novel-miR-1289, and *Fbln2* (Fig. 6D).

## Discussion

**Fig. 7:**
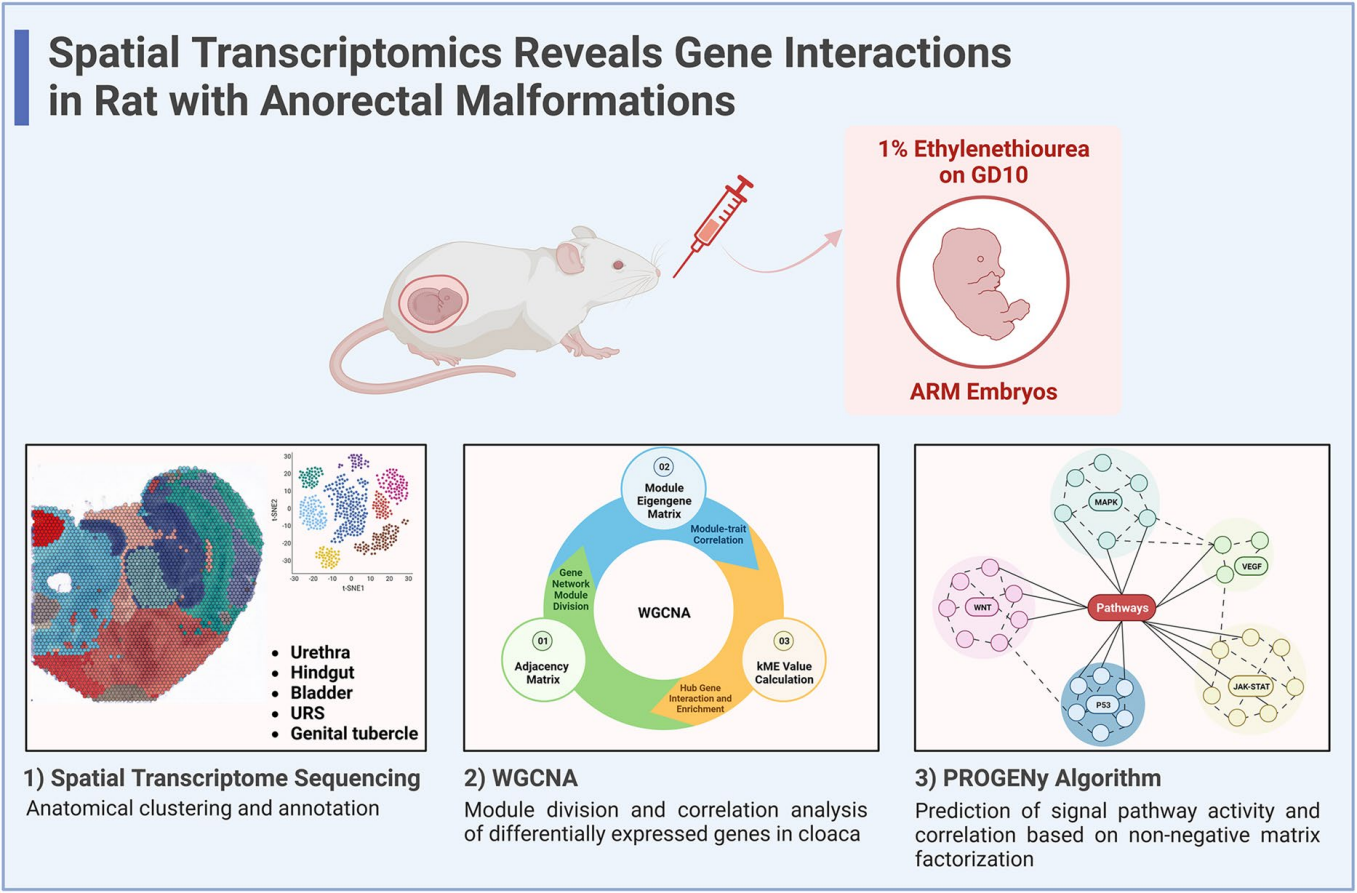
Graphical summary depicts the sequencing of normal and ARM rat embryos on GDs 14–16 using spatial transcriptomics. Anatomical clustering and annotation were performed, and gene expression characteristics and signaling pathway activities were explored through various bioinformatics analysis methods, including WGCNA and PROGENy

In-depth research on ARM pathogenesis is still lacking, and while surgical interventions can reconstruct normal anatomical structures, exploring the molecular biological changes underlying these malformations could significantly advance prenatal diagnosis and pregnancy management. In this study, through the spatial transcriptome sequencing of the critical period of rat embryonic anorectal development (GDs 14–16), we obtained the transcriptome data with anatomical structure specificity. Based on WGCNA analysis and other methods, we revealed the relationship between gene modules and location structure as well as gestation days and explored the timing and localization of differential genes in biological function, thus, demonstrating the spatiotemporal expression characteristics of different genes. Additionally, the spatiotemporal characteristics of common signaling pathway activity in ARM embryos were also mentioned, and the synergy or opposite trends between signaling pathway correlations provided a new perspective on pathway interaction. Finally, we integrated the entire transcriptome dataset to construct a regulatory network for non-coding RNAs (Fig. 7).

WGCNA serves as a valuable tool for data exploration and gene selection. It allows us to delve into the module structures within networks, measure relationships between genes, modules, and gene-module interactions, and rank genes or modules (Niemira et al. 2019; Lin et al. 2021). During the WGCNA analysis, genes within the grey module, representing a small fraction of all genes, are excluded from subsequent analyses. This is because these genes exhibit weak co-expression relationships with others or have distinct expression patterns. While there may be disease-related genes in the grey module, these genes are weakly correlated with cloaca development and thus cannot be detected through WGCNA. In this study, there was a commonality between the normal and ARM groups, the gene modules of GD14 and GD15 displayed a consistent trend of positive correlation, whereas the gene modules of GD16 showed the opposite. According to previous theoretical foundations, this may be because of the continuous downward extension of the URS during GDs 14–15, causing the gradual separation of the hindgut and the urethra. This is a dynamic and continuous process, and regardless of whether the hindgut and urethra are successfully separated, this process ends before GD16 (Qi et al. 2000; Tang et al. 2018). Therefore, based on the characteristic module analysis of clusters 0–4, the gene modules that showed positive correlation trends at GD14 and GD15 may be the key effector molecules that promote normal or ARM development.

Betweenness centrality is a crucial concept in network analysis, used to quantify the extent to which a node serves as a mediator in the connections between other nodes. When applied to gene expression networks or protein-protein interaction networks, betweenness centrality is utilized to evaluate the importance of genes or proteins within the regulatory framework. Nodes with high betweenness centrality typically exert strong control and influence in the network, as they lie on many shortest paths between pairs of nodes, acting as critical bridges for information transfer (Nithya et al. 2024; Zito et al. 2021; Heryanto et al. 2022). Traditional gene enrichment analysis and pathway prediction methods, such as GO or KEGG, typically analyze differentially expressed genes at the transcriptional level by associating them with corresponding protein signals, which overlooks the effect of post-transcriptional modifications. PROGENy can infer pathway activity from gene expression under different conditions and improve the accuracy of the predicted results (Holland et al. 2020). From the PROGENy prediction results, Wnt signaling was mainly concentrated in the genital tubercle region and was also present in the hindgut and URS. Previous studies have suggested that Wnt signaling and ARM are closely related, and genes such as *Wif1*, *Wnt5a*, and *β-catenin* play important roles in cloaca development (Ng et al. 2014; Li et al. 2011; Miyagawa et al. 2014).

In recent years, the mechanisms of some non-coding RNAs (ncRNAs) such as miR-92a-2-5p, miR-141-3p, and circ0005139 involved in the occurrence of ARM have gradually been revealed (Long et al. 2020; Wang et al. 2022; Liu et al. 2020). For example, circJag1 is upregulated in ARM and promotes the apoptosis of intestinal epithelial cells by inhibiting the classical Wnt/β-catenin pathway through the miR-137-3p/Sox9 axis, which leads to the fusion failure of the URS and CM, and the occurrence of ARM (Li et al. 2023). As important regulators of gene expression in organisms, ncRNAs play an indispensable role in ARM. Therefore, combining spatial transcriptomics and whole-transcriptome sequencing data to construct a structurally specific ncRNA binding network can indirectly screen out ncRNA molecules that may play potential biological roles in the hindgut or URS region. Comparing genes in the ceRNA network with the selected differentially expressed genes aims to identify their intersection, elucidating the portion of differentially expressed genes present in the ceRNA network. Subsequent utilization of network analysis tools allows for visualization and analysis, facilitating the recognition of key nodes and interaction relationships within the network. Associating differentially expressed genes with the ceRNA network provides a means to better comprehend the intricate interactions among these genes within the cellular context, offering valuable insights for a more profound exploration into the mechanisms underlying ARM occurrence.

This study has certain limitations. Firstly, the gene expression data obtained through spatial transcriptomics are based on the mRNA level, and their protein-level expression, localization, and function require further validation. Secondly, this study primarily employed bioinformatics methods to predict potential gene interactions and signaling pathway activities, and the molecular mechanisms underlying ARM development warrant further investigation. Lastly, this study only describes potential genes and signals during rat cloacal development. Extending and extrapolating these data results to other species require more in-depth exploration.

## Conclusions

In conclusion, this study employed spatial transcriptomics technology to sequence normal and ARM rat embryos during GDs 14–16. Through various bioinformatics analyses to decipher the gene interaction characteristics primarily in the cloacal region. WGCNA was utilized to identify gene modules linked to the development of diverse anatomical structures in the cloacal region, and these modules demonstrated synchronized activity on both GD14 and GD15. Hub genes were identified through WGCNA and protein-protein interaction networks, validating the low expression of proteins such as Pcsk9, Hmgb2, and Sod1 in the ARM hindgut on GD15. The function of hub genes not only correlated with the predicted signaling pathway activities by the PROGENy algorithm but also allowed predictions of ceRNA relationships within the network constructed from whole-transcriptomic data. The multifaceted bioinformatics analyses in this study aim to deepen our understanding of molecular interactions and changes in signaling pathway activities associated with ARM.

## Data Availability

The datasets used in this study are available from the corresponding author upon request.
